# MYEF2: an immune infiltration-related prognostic factor in IDH-wild-type glioblastoma

**DOI:** 10.18632/aging.204939

**Published:** 2023-08-08

**Authors:** Yunxiao Zhang, Yunyu Wen, Jing Nie, Tong Wang, Gang Wang, Qiaoping Gao, Yongfu Cao, Hai Wang, Songtao Qi, Sidi Xie

**Affiliations:** 1 Department of Neurosurgery, Nanfang Hospital, Southern Medical University, Guangzhou 510515, Guangdong, PR China; 2 Department of Neurosurgery, The Third Hospital of Mianyang (Sichuan Mental Health Center), Mianyang 621000, Sichuan, PR China; 3 Department of Medical Quality Management, Nanfang Hospital, Southern Medical University, Guangzhou 510515, Guangdong, PR China; 4 Neurosurgery, Key Laboratory of Biological Targeting Diagnosis, Therapy and Rehabilitation of Guangdong Higher Education Institutes, The Fifth Affiliated Hospital of Guangzhou Medical University, Guangzhou 510700, Guangdong, PR China

**Keywords:** MYEF2, weighted gene co-expression network analysis (WGCNA), immune infiltration, RNA binding proteins (RBPs), glioblastoma

## Abstract

Glioblastoma (GBM) is the most malignant and prevalent primary brain tumor. In this study, weighted gene coexpression network analysis (WGCNA) was performed to analyze RNA binding protein (RBP) expression data from The Cancer Genome Atlas (TCGA) for the IDH-wild type GBM cohort. The CIBERSORT algorithm quantified the cellular composition of immune cells and was used to identify key modules associated with CD8+ T cell infiltration. Coexpression networks analysis and protein-protein interaction (PPI) network analysis was used to filter out central RBP genes. Eleven RBP genes, including MYEF2, MAPT, NOVA1, MAP2, TUBB2B, CDH10, TTYH1, PTPRZ1, SOX2, NOVA2 and SCG3, were identified as candidate CD8+ T cell infiltration-associated central genes. A Cox proportional hazards regression model and Kaplan-Meier analysis were applied to identify candidate biomarkers. MYEF2 was selected as a prognostic biomarker based on the results of prognostic analysis. Flow Cytometric Analysis indicated that MYEF2 expression was negatively correlated with dysfunctional CD8+ T cell markers. Kaplan–Meier survival analysis (based on IHC staining) revealed that GBM patients with elevated MYEF2 expression have a better prognosis. Knockdown of MYEF2 in GBM cells via *in vitro* assays was observed to promote cell proliferation and migration. Our study suggests that MYEF2 expression negatively correlates with T cell exhaustion and tumor progression, rendering it a potentially valuable prognostic biomarker for GBM.

## INTRODUCTION

Glioblastoma (GBM) is the deadliest and most aggressive tumor in the adult central nervous system. Standardized treatment combining surgical resection with maximum safety range, radiotherapy, adjuvant temozolomide (TMZ) chemotherapy and tumor-treating fields (TTF) results in median survival time of adult patients with GBM 18 months after diagnosis [1]. The fifth edition of the WHO Classification of Tumors of the Central Nervous System (WHO CNS5) emphasizes the important role of molecular biomarkers in the diagnosis of GBM and defines GBM as an IDH-wild-type diffuse and astrocytic glioma in adults accompanied by microvascular proliferation or necrosis or TERT promoter mutation or EGFR gene amplification or +7/−10 chromosome copy number changes (GBM, IDH wildtype) to distinguish it from astrocytoma, IDH-mutant [2, 3]. In addition to these biomarkers, others, such as MGMT promoter methylation, have also been confirmed to be associated with the prognosis of GBM [4, 5]. However, more molecular biomarkers should be identified to benefit the diagnosis and treatment of GBM patients.

The tumor microenvironment, which contains many different non-cancerous cell types in addition to cancer cells, has a crucial role in cancer growth, metastasis, and response to treatment. Despite general immunosuppression in the normal brain, glioma cells can secrete numerous chemokines, cytokines and growth factors that promote infiltration of various cells, including a range of immune cells into the tumor. The presence of T cells in cancer lesions has long been known to be correlated with better patient prognosis in various human malignancies, e.g., within primary GBM, elevated numbers of intratumoural cytotoxic T cells (CD3+CD8+) significantly correlated with a better survival [6]. However, researchers recently realized the existence of high diversity in the activation and dysfunctional states of the T cells in human cancer lesions. T cells can display features of ‘dysfunction’ or ‘exhaustion’, that is a hallmark of GBM. Dysfunctionality of T cells in human tumor is characterized by the increased cell surface expression of inhibitory receptors, including PDCD1, LAG3, CTLA4, TIGIT and HAVCR2, and a reduced capacity of the cells to carry out classical CD8+ T cell effector functions [7, 8]. This may be one of the reasons that antagonizing or blocking PD-1 and CTLA-4, which is well-recognized FDA-approved anticancer strategies aimed at improving T cell function in multiple malignancies, have shown only limited efficacy in GBM [9]. Thus, identification of CD8+ T cell-related factors will help understanding its differentiation in human tumors, and offer new possibilities for patient stratification and therapeutic intervention.

RNA-binding proteins (RBPs) have been verified to play important roles in tumor progression [10]. RBPs are involved in RNA posttranslational regulation, including RNA splicing, localization, stability, degradation, polyadenylation and translation [11, 12]. Alterations in RBP expression and activation contribute significantly to the development of various tumor, including GBM [13]. However, it has not yet been determined whether there are RBPs associated with GBM immune infiltration.

In this study, we constructed an RBP gene network based on weighted gene coexpression network analysis (WGCNA) and recognized immune infiltration-related gene modules based on gene expression data from The Cancer Genome Atlas (TCGA) IDH-wild-type GBM cohort. We identified and validated CD8+ T cell-associated hub genes using the TIMER and TISIDB databases. Furthermore, we applied the Cox proportional hazards regression model and Kaplan–Meier analysis to filter prognostic biomarkers. These works contribute to the development of new biomarkers for immunotherapy and prognosis of GBM patients.

## MATERIALS AND METHODS

### Data acquisition and processing

RNA sequencing profiles and clinic-pathological information of GBM samples were collected from TCGA (https://xenabrowser.net, accessed on 28th September, 2020). Samples with IDH mutation were filtered out according to the SNP mutation information, while control samples were filtered out according to the sample information. Finally, only samples of IDH wildtype GBM were included (*n* = 164) (Supplementary Table 1).

RNA sequencing data were normalized using R-package limma [14]. After excluding genes with missing values, 20530 genes were finally obtained from the TCGA dataset. Among these, 3563 genes were identified as RBP genes according to the published research [15].

### Evaluation of tumour-infiltrating immune cells

The proportions of tumor-infiltrating immune cells in the TCGA IDH-wild-type GBM samples (*n* = 164) were estimated using the R-package CIBERSORT [16]. CIBERSORT employs a support vector regression and deconvolution algorithm to estimate the abundances of specific immune cell types based on gene expression data, utilizing a set of reference gene expression values (547 genes).

### Construction of the weighted gene coexpression network

The weighted gene coexpression network was constructed using the expression values of the 3563 RBP genes with the R-package WGCNA [17]. The GBM samples (*n* = 164) were clustered using average linkage and Pearson’s correlation coefficients. A soft threshold (power) was then determined to create the weighted adjacency matrix and topological matrix. Next, a dynamic hybrid cutting method was applied, defining a module minimum size of 30 and a dissimilarity threshold between genes of less than 0.2. Gene significance (GS) was computed as the absolute correlation coefficients between genes and sample traits. Additionally, the module eigengene, representing the leading principal component of the module’s expression matrix, was extracted. The absolute correlation coefficient was utilized to assess its relationship with T cell infiltration levels (module significance, MS), enabling the selection of the hub module with the highest MS and a *p*-value below 0.05.

### Functional enrichment analysis

The function of genes within the identified hub module was determined using the R-package clusterProfiler [18]. Gene Ontology (GO) [19] and Kyoto Encyclopedia of Genes and Genomes (KEGG) [20] were utilized. Significantly enriched functions were identified using an adjusted *p*-value threshold of <0.05 (false discovery rate, FDR).

### Identification of hub genes in the hub module

The selection of candidate hub genes in the hub module was based on their modular connectivity and relationship with clinical traits. Module connectivity was defined as the absolute value of the Pearson’s correlation coefficients between genes and the module eigengene (module membership). As mentioned earlier, the relationship between each gene and the clinical trait was indicated using gene significance (GS). Genes with a module membership >0.7 and a GS >0.2 were considered as candidate hub genes. Moreover, a protein-protein interaction (PPI) network was constructed using all the genes within the hub module. The Search Tool for the Retrieval of Interacting Genes (STRING; https://string-db.org/) database [21] was employed to identify the central nodes in the PPI network based on node connectivity (>15). The PPI network was presented by Cytoscape (https://cytoscape.org, accessed on 28th September, 2020) [22]. For further analyses, a Venn analysis was conducted using an online tool (http://bioinformatics.psb.ugent.be/webtools/Venn) to extract hub genes in an overlap between candidate hub genes in the module and central nodes in the PPI network.

### Validation of hub genes’ relation with tumor-infiltrating immune cells

To further investigate the relationship between hub genes and tumor-infiltrating immune cells, Spearman correlations were performed on the expression data of the hub genes in the TIMER database (https://cistrome.shinyapps.io/timer) and in the TISIDB database (http://cis.hku.hk/TISIDB/index.php). Hierarchical cluster diagrams and scatter plots were created. *P*-values < 0.05 indicates statistical significance.

### Survival analysis

Univariate and multivariate Cox regressions (*n* = 164) were performed to assess the association of gene expression and patient prognosis, and Kaplan–Meier curve (*n* = 164) was created with log-rank test to detect difference of survival curves. The best cut-off value of gene expression was determined by the surv_cutpoint function in the R-package “survminer”. Two R packages, “survival” and “survminer”, were used to further visualize the risk curve during analysis and facilitate reading and analysis. *P*-values < 0.05 indicates statistical significance.

### Gene set enrichment analysis (GSEA)

GSEA is an analytical method of determining whether a set of specific functional gene sets exhibits statistically significant differences between two groups [23]. See Supplementary Materials and Methods available online for details.

### Patients and tissue samples

The study included patients who were independently diagnosed with primary GBM by two pathologists in a double-blinded manner, in accordance with the criteria of the 2021 WHO classification. These patients had undergone standard surgery at the Department of Neurosurgery, Nanfang Hospital, located in Guangzhou City, Guangdong Province, China, between 2016 and 2021, without any prior radiotherapy or chemotherapy. The Ethics Committee of Southern Medical University approved the study, and all enrolled patients provided informed consent.

### RNA isolation and qRT-PCR

14 cases of GBM tissues were used for qRT-PCR experiments to measure MYEF2 mRNA expression. See Supplementary Materials and Methods available online for details.

### GBM tissue single-cell dissociation

See Supplementary Materials and Methods available online for details.

### Flow cytometric analyses

The cells derived from GBM tissues (*n* = 14) were thoroughly washed and subsequently stained with a panel of antibodies for a duration of 15 minutes in a dark environment. The stained cells were then assessed using flow cytometry. The antibody cocktail consisted of six different antibodies: CD45 BUV395 (BD Biosystems, Catalog No. 569489), CD3 Pe-Cy7 (eBioscience, Catalog No. 25–0037-42), CD8 Pacific Blue (BD Biosystems, Catalog No. 558207), PD-1 BV650 (BD Biosystems, Catalog No. 752738), HAVCR2 AF700 (eBioscience, Catalog No. 56-3109-42), and LAG3 AF647 (BD Biosystems, Catalog No. 565717). Each antibody was diluted to achieve a 1:40 ratio with the buffer. Subsequently, all the samples were analyzed using FACS Aria II (BD Bioscience), and the obtained data were further analyzed using FlowJo software.

### Immunohistochemistry (IHC)

47 cases of GBM tissues with matching clinical data (Supplementary Table 2) were used for IHC experiments to study altered MYEF2 protein expression as previously described [24]. See Supplementary Materials and Methods available online for details.

### Cell lines and culture

The U87MG human GBM cell line was obtained from American Type Culture Collection (ATCC: Rockville, MD, USA). The NFHGBM primary human GBM cell line was derived and cultured from a GBM patient at Nanfang Hospital [25].

### Transient knockdown of MYEF2 in GBM cells

Cells were transfected with chemosynthetic siRNAs (Gene Pharma Biotechnology Co., Shanghai) using Lipofectamine 2000 reagent (Invitrogen, Cat# 11668) according to the manufacturer’s protocol. The sequences of the siRNAs are shown in Supplementary Table 3.

### EdU assays

EdU assays (*n* = 6 per group) were carried out as previously described [25]. See Supplementary Materials and Methods available online for details.

### *In vitro* migration assays

*In vitro* migration assays (*n* = 5 per group) were carried out as previously described [26]. See Supplementary Materials and Methods available online for details.

### Statistical analysis

All *in vitro* experiments were repeated at least 3 times. Statistical analyses were performed using R software version 3.5.0, SPSS statistical software version 20.0 and GraphPad Prism software version 7.0. To test the associations of clinical characteristics (i.e., gender and age) with survival status and MYER2 expression, chis-square test was used. To detect the difference of expression of hub genes among different age groups (young, middle and old) and between females and males, Kruskal-Wallis test was conducted. With regard to *in vitro* experiments, mean and standard deviation (SD) were calculated in two cell lines before and after knockdown of MYEF2, and compared by Student’s *t* tests or one-way ANOVA. *P* < 0.05 was considered statistically significant.

## RESULTS

### Data preprocessing and evaluation of tumor-infiltrating immune cells (TIICs)

**Figure 1:**
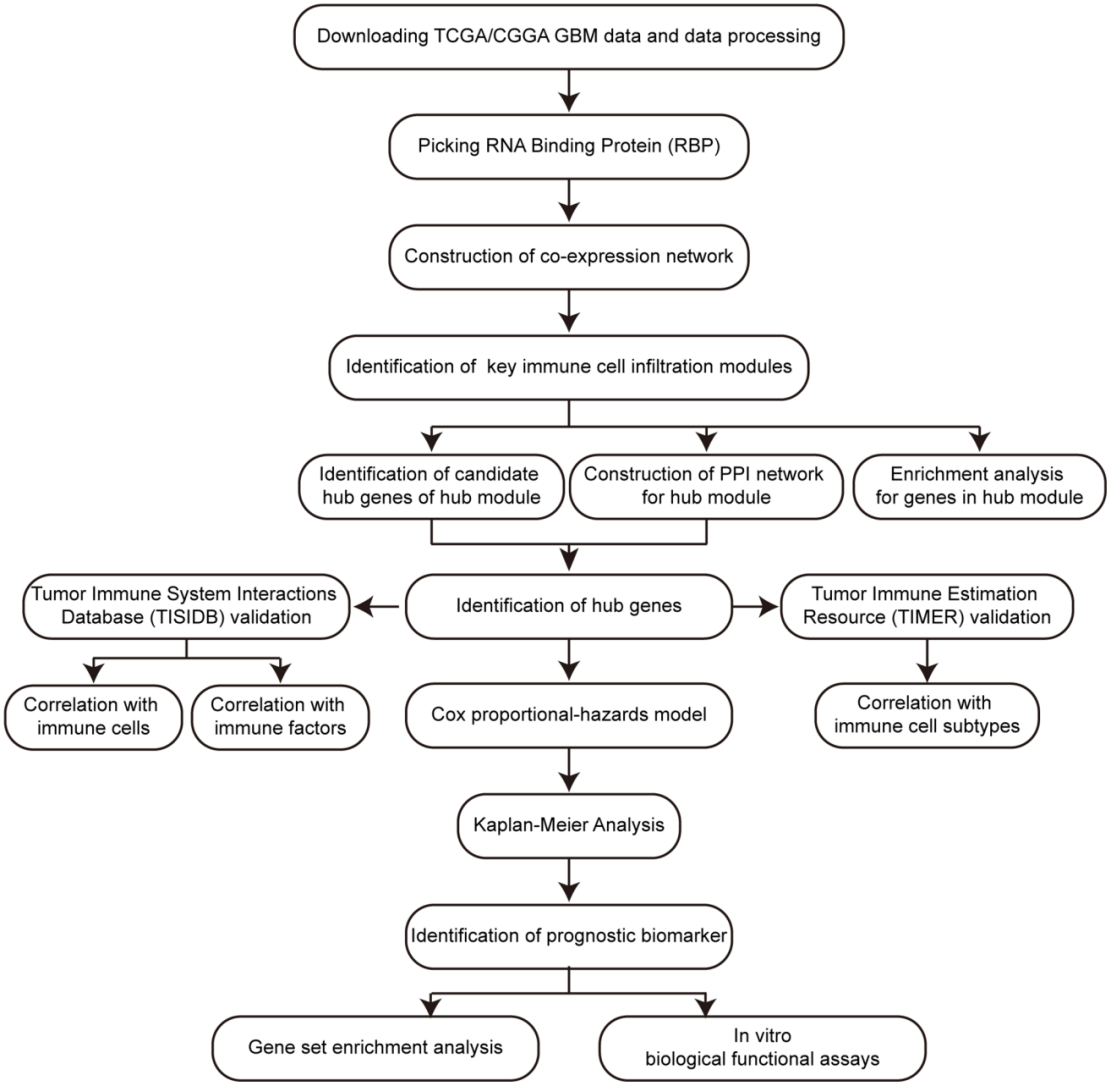
Workflow of this research.

The research strategy is shown in Figure 1.

**Figure 2:**
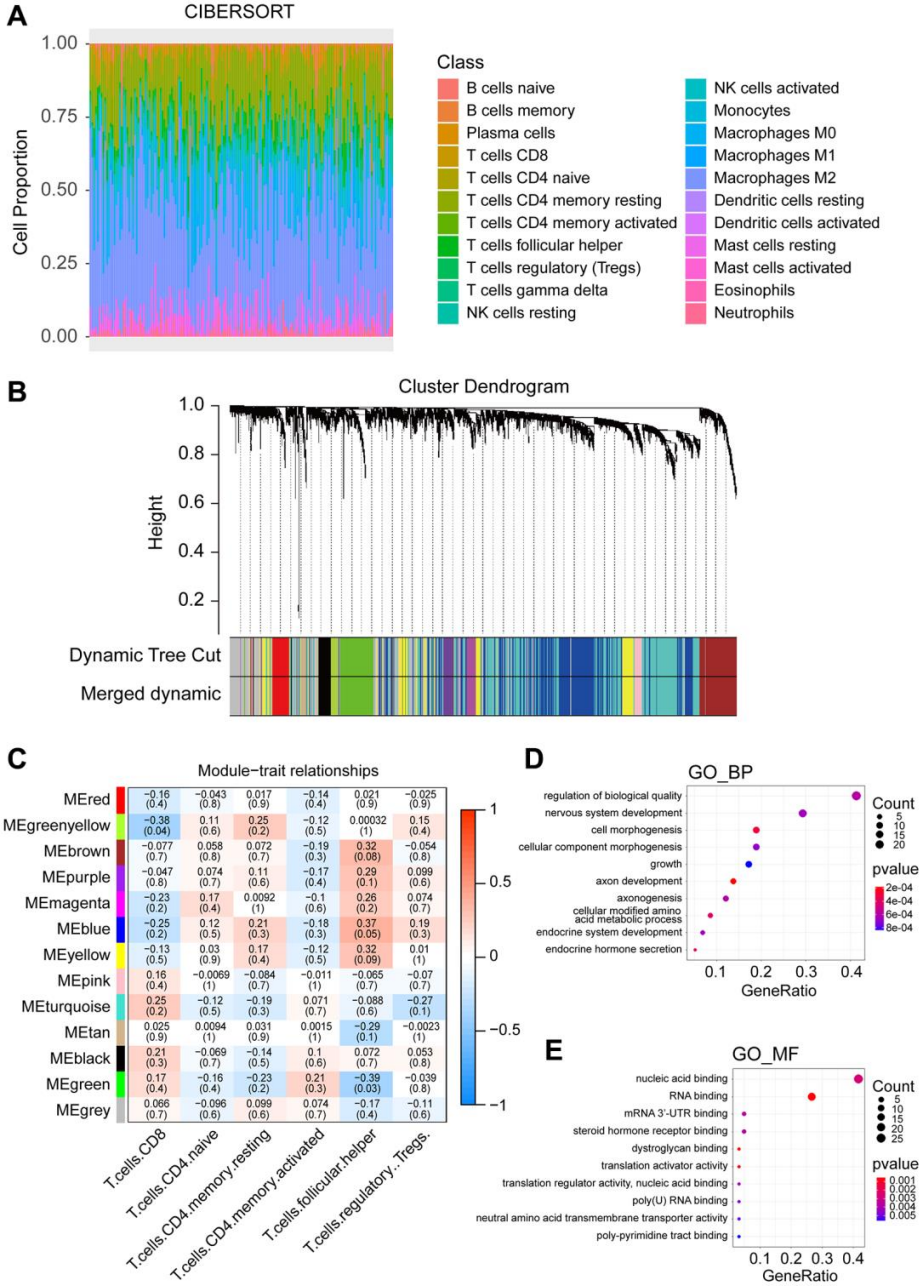
**Constructing the weighted co-expression network based on RBP genes and identifying key modules.** (**A**) Infiltration ratio of 22 immune cells in DH wild-type GBM samples. (**B**) Hierarchical cluster analysis was conducted to detect co-expression clusters of the RBP genes. Each colour represents a module. (**C**) Correlation between different gene modules and T cell infiltration ratio. The upward numbers in the boxes represent Pearson R values, and the numbers in the brackets represent *P*-values. (**D**) Top ten enrichment items of GO_BP analysis. (**E**) Top ten enrichment items of GO_MF analysis.

In the GBM cohort from TCGA, 20530 genes of 164 samples were finally retained. Furthermore, expression of 3563 RBP genes were screened in this cohort [15]. Based on CIBERSORT, proportions of 22 types of immune cells in the 164 samples were shown in Figure 2A.

### Weighted coexpression network construction

Using soft threshold = 4 (scale-free R^2^ = 0.85) (Supplementary Figure 1A), a coexpression network was constructed based on the 3563 RBP genes and thirteen modules were identified (Figure 2B). The previously calculated immune infiltration proportions of seven types of T cells were selected as trait data for WGCNA, and the clustering dendrogram of the 164 IDH-wild-types GBM samples was shown in Supplementary Figure 1B.

### Identification of a hub key module related to T cell infiltration and GO enrichment analysis

We constructed a correlation network according to the coexpression module constructed by WGCNA and the T cell infiltration proportions of the samples (Figure 2C). Since we focused on CD8+ T cell infiltration, we selected the greenyellow module that showed the highest absolute correlation coefficient with CD8+ T cell infiltration (r = −0.38, *P* = 0.04) as the key module for follow-up research. Genes included in the greenyellow module were subsequently analyzed for functional enrichment in GO. The top five enrichment terms of biological process (BP) were regulation of biological quality, nervous system development, cell morphogenesis, cellular component morphogenesis and growth (Figure 2D). In the molecular function (MF) category, the top ten enrichment terms were all associated with RNA binding and nucleic acid binding (Figure 2E).

### Identification and validation of hub genes

**Figure 3:**
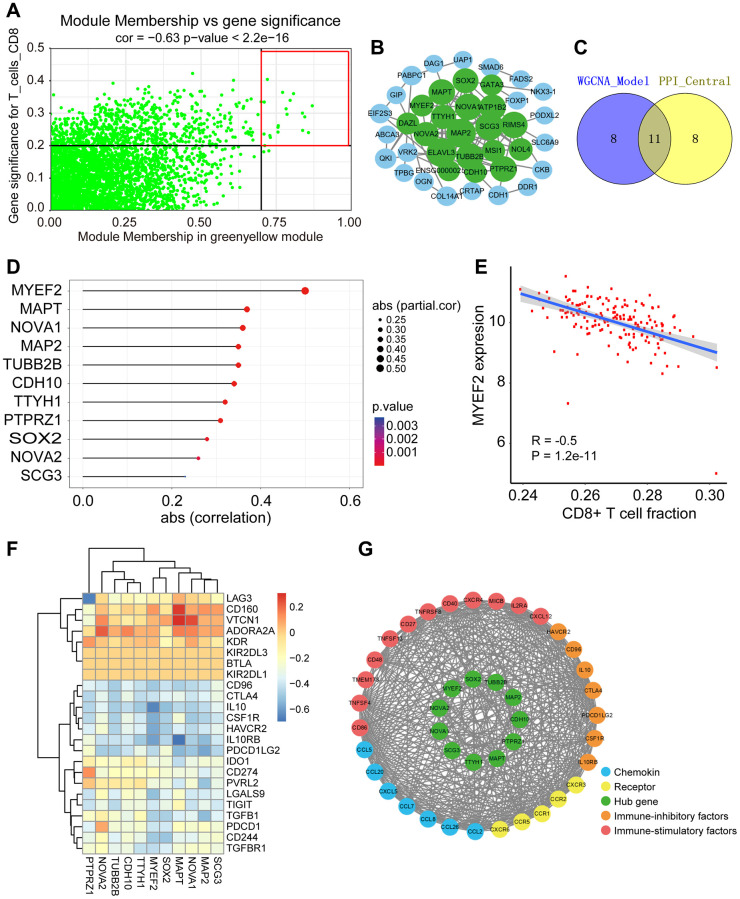
**Identification of hub genes and evaluation of correlation with immune infiltration of hub genes.** (**A**) Scatter plot of the genes in the greenyellow module. Each green dot represents a gene, and dots within the red box indicate genes of Module Membership >0.7 and Gene Significance >0.2. (**B**) PPI network of genes from the greenyellow module. Larger size of the node means higher number of connected nodes. Green nodes represent central nodes with more than 15 connections. (**C**) Venn plot of the overlap genes between co-expression and PPI networks. (**D**) Scatter plot of the correlation between 11 hub genes expression and CD8+ T cell infiltration level in TIMER database. (**E**) Scatter plot of the correlation between MYEF2 expression and CD8+ T cell infiltration level in TIMER database. (**F**) Heatmaps of spearman correlations between hub genes expression and expression of immune-inhibitory factors in TISIDB database. (**G**) PPI network of GBM immune microenvironment and the 11 hub genes.

According to the threshold criteria (module membership >0.7 and GS >0.2, q.weighted <0.01), 19 candidate hub genes were identified from the greenyellow module (Figure 3A, Supplementary Table 4). Furthermore, the PPI network of the coexpressed genes from the greenyellow module was built (Figure 3B), and another 19 candidate hub genes were identified (connectivity >10). Finally, 11 hub genes (MYEF2, MAPT, NOVA1, MAP2, TUBB2B, CDH10, TTYH1, PTPRZ1, SOX2, NOVA2 and SCG3), which were both in the two hub gene sets, were obtained (Figure 3C).

Further analyses in the TIMER database revealed negative correlations between the expression of all 11 hub genes and the level of CD8+ T cell infiltration (Figure 3D). As depicted in Figure 3E, we presented a scatter plot illustrating the correlation between MYEF2 expression and CD8+ T cell infiltration levels, while the remaining genes were depicted in Supplementary Figure 2. Additional analyses in the TISIDB database showed that the hub genes were negatively correlated with activated CD8+ T cells (Act CD8) and effector memory CD8+ T cells (TEM CD8) (Supplementary Figure 3A). These findings supported that these 11 hub genes actively contribute to the immune microenvironment by strongly negatively associating with CD8+ T cell infiltration.

Next, we analyzed the correlations between expression of hub gene and immune factors in the TISIDB database, including immune-inhibitory factors, immune-stimulatory factors, chemokines and receptors (Supplementary Figure 3B–3D). Specifically, we observed a negative correlation between MYEF2 expression and several immune-inhibitory factors, including LAG3, PDCD1, PDCD1LG2, CTLA4, TIGIT and HAVCR2, which were characteristics of dysfunctional CD8+ T cells (Figure 3F). Utilizing an average absolute Spearman correlation coefficient greater than 0.35, we identified 33 immune-related factors that exhibited strong associations with the 11 hub genes. Based on these 33 immune-related factors and the 11 hub genes, an immune infiltration interaction network was constructed using STRING and visualized through Cytoscape (Figure 3G). Among these 33 immune-related factors, HAVCR2, CTLA4 and PDCD1LG2 were also found to be highly correlated with the 11 hub genes including MYEF2.

### Determination of clinical characteristics

Next, we investigated the association between 11 hub genes and GBM patient clinical characteristics in the TCGA database. In the TCGA IDH-wild-type GBM cohort, the expression of most of the hub genes showed no significant difference between patients of different ages. In contrast, MAP2 expression decreased with patient age, while SCG3 displayed the highest expression in middle-aged patients (Supplementary Figure 4A). In addition, none of the hub genes were correlated with patient sex (Supplementary Figure 4B).

### Identification of prognostic biomarkers

**Figure 4:**
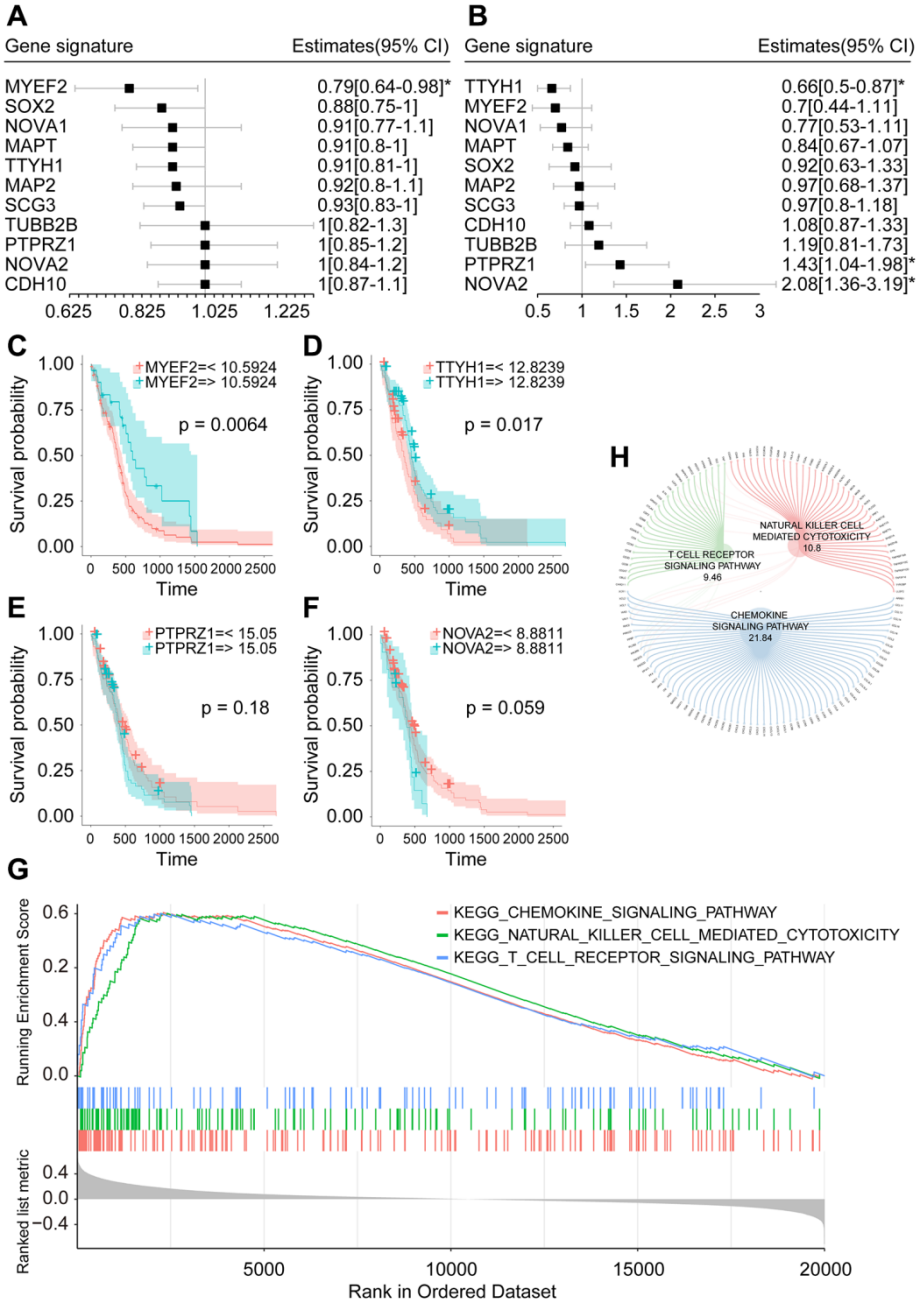
**Prognostic analysis of hub genes.** (**A**) Forest plot of univariate Cox analysis of hub genes. (**B**) Forest plot of multivariate Cox analysis of hub genes. (**C**) Overall survival analysis of MYEF2. (**D**) Overall survival analysis of TTYH1. (**E**) Overall survival analysis of PTPRZ1. (**F**) Overall survival analysis of NOVA2. (**G**) Plot of the top three enriched pathways in GSEA analysis of MYEF2. (**H**) Circle diagram of core genes in the top three enriched pathways in GSEA analysis of MYEF2. Larger circle corresponding to each gene represents larger rank metric score value.

Through univariate Cox model, we found that only MYEF2 was correlated with patient prognosis (HR = 0.79, *P* < 0.05) (Figure 4A). However, multivariate Cox analysis showed that three genes, TTYH1 (HR = 0.66, *P* < 0.05), PTPRZ1 (HR = 1.43, *P* < 0.05) and NOVA2 (HR = 2.08, *P* < 0.05), were associated with patient prognosis (Figure 4B). We analyzed these genes by Kaplan–Meier analysis and found that patients with higher MYEF2 expression exhibited better survival outcomes (*P* = 0.0064) (Figure 4C); likewise, those with higher TTYH1 expression exhibited better survival outcomes (*P* = 0.017) (Figure 4D). However, the expression levels of PTPRZ1 and NOVA2 were not correlated with the survival outcomes of patients (Figure 4E and 4F). Since MYEF2 showed the highest correlation coefficient with CD8+ T cells, as shown in Figure 3D, 3E, we selected MYEF2 as a candidate prognostic biomarker for further analysis.

### Gene set enrichment analysis (GSEA) of MYEF2

According to the previously calculated expression threshold of MYEF2 (10.5924), the samples of the TCGA IDH-wild-type GBM cohort were divided into a high expression group and a low expression group for GSEA. The enrichment results showed that a total of 41 immune-related pathways were statistically significantly enriched in the low MYEF2 expression group (*p*.adj < 0.05). The top three enriched pathways were “chemokine signaling pathway”, “natural killer cell mediated toxicity” and “T cell receiver signaling pathway” (Figure 4G). We show the core genes in these three pathways in Figure 4H.

### MYEF2 expression negatively correlates with T cell exhaustion and tumor progression of GBM

**Figure 5:**
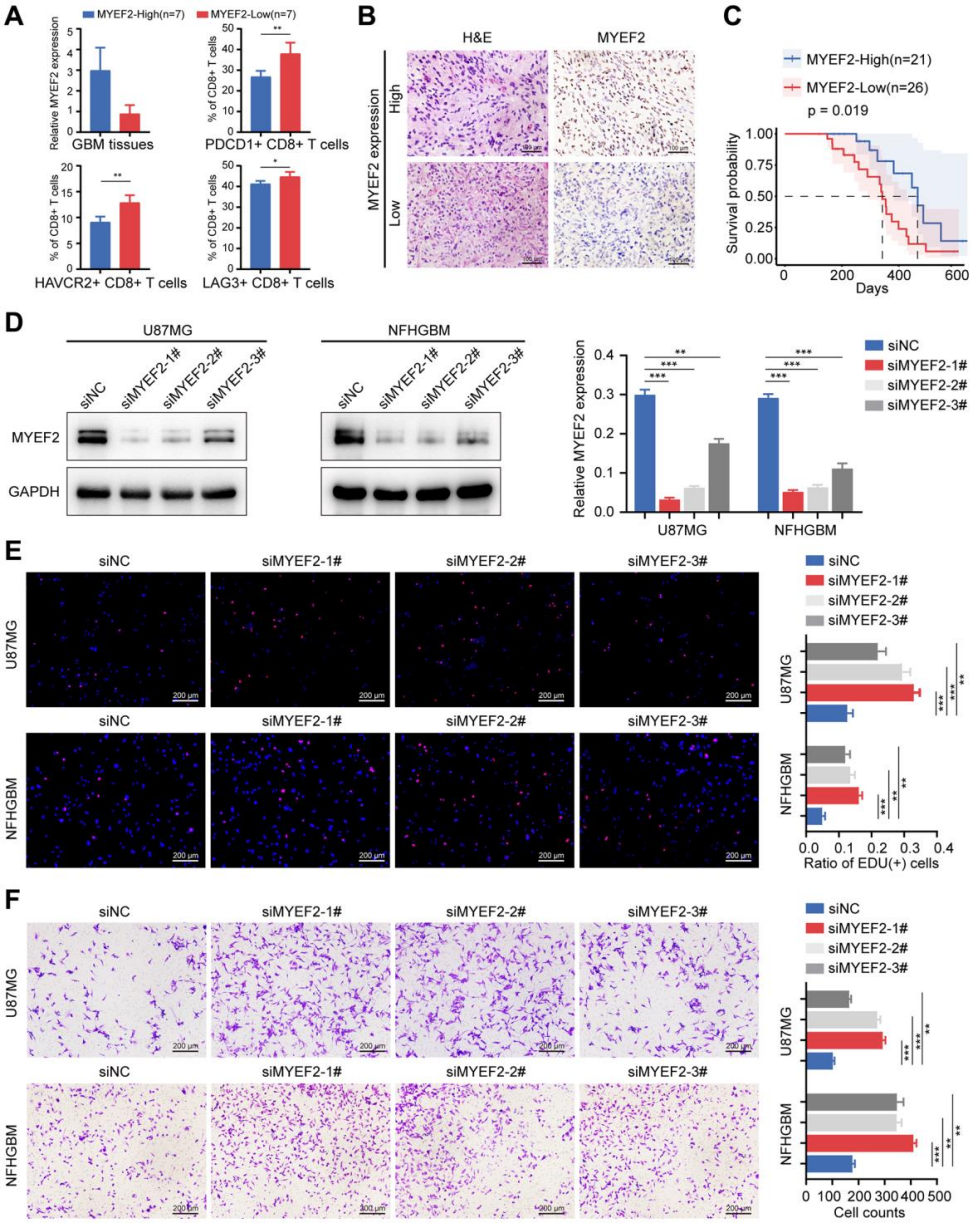
**MYEF2 negatively correlates with GBM CD8+ T cells exhaustion and tumor progression.** (**A**) Bar graph of the qRT-PCR of MYEF2 expression in GBM tissues (*n* = 14) and percent of PDCD1, HAVCR2 and LAG3 expression on CD8+ T cell from MYEF2-High and MYEF2-Low samples as measured by flow cytometry. ^*^P < 0.05, ^**^P ≤ 0.001. (**B**) Representative images of MYEF2 immunohistochemical staining of GBM tissues. (**C**) Kaplan–Meier survival curve of NFH-GBM patients stratified by MYEF2 expression (according to IHC data). (**D**) Western blot assays showing MYEF2 knockdown efficiency in U87MG and NFHGBM cells. (**E**) EdU assay showing different cell proliferation rates in siMYEF2- and siNC-treated U87MG and NFHGBM cells. Error bars represent the SD of repeats of each cell. ^**^P < 0.01, ^***^P < 0.001. (**F**) Transwell assay showing cell migration capacity in siMYEF2- and siNC-treated U87MG and NFHGBM cells. Error bars represent the SD of repeats of each cell. ^**^P < 0.01, ^***^P < 0.001.

To explore the relationship between MYEF2 expression, as determined by qRT-PCR, and T cell exhaustion, we utilized flow cytometric approaches to analyze the expression of biomarkers for dysfunctional CD8+ T cells, specifically PDCD1, HAVCR2, and LAG3, in 14 primary GBM tissues. Our analysis revealed that elevated MYEF2 expression was associated with reduced T cell exhaustion of GBM, as illustrated in Figure 5A. Next, we performed immunohistochemical staining in tumor specimens from 47 GBM patients (Figure 5B). Patient survival analysis indicated a clear positive correlation between MYEF2 protein expression level (according to IHC staining score) and the overall survival time in GBM patients (Figure 5C). Collectively, MYEF2 served as a favourable prognostic marker in GBM.

Next, we used three distinct siRNAs to knock down MYEF2 expression in the GBM cell line U87MG and the primary GBM cell line NFHGBM. The knockdown efficiency was shown in Figure 5D. Three siRNAs effectively knocked down MYEF2 expression in both cell lines. Further *in vitro* cell biological experiments revealed that MYEF2 silencing by siRNAs transfection significantly elevated the proliferation of both U87MG and NFHGBM cells (Figure 5E), and promoted migration capacity of both cell lines (Figure 5F).

## DISCUSSION

GBM is the most malignant primary tumor in the adult central nervous system. Although there are many significant research breakthroughs in the immunotherapy of GBM, it has not yet been effective enough in clinical GBM treatment applications. RBPs play crucial roles in GBM progression, but little is known about their association with immune infiltration and key genes in GBM progression.

In this study, we constructed an RBP gene network by WGCNA based on the TCGA IDH-wild-type GBM cohort and identified a key gene module negatively associated with CD8+ T cell infiltration. Subsequently, we filtered out 11 hub genes (MYEF2, MAPT, NOVA1, MAP2, TUBB2B, CDH10, TTYH1, PTPRZ1, SOX2, NOVA2 and SCG3) from this gene module. Further analysis based on the TIMER and TISIDB databases revealed negative correlations between these hub genes and CD8+ T cells separately, indicating the crucial role of these genes in shaping the GBM immune microenvironment and escaping immune surveillance. Moreover, the Cox proportional hazards regression model and Kaplan–Meier analysis demonstrated that the prognosis of patients with higher expression of MYEF2 or TTYH1 was significantly better. Thus, MYEF2 and TTYH1 were selected as potential CD8+ T cell infiltration-related RBP biomarkers for the prognosis prediction of GBM.

The MYEF2 gene encodes a protein mainly expressed in brain tissues. It binds to the promoter of the myelin basic protein gene (MBP) and represses its transcription, leading to oligodendrocyte progenitor cell (OPC) differentiation disorder and demyelination disease [27, 28]. MYEF2 has also been reported to be associated with haematopoietic stem cell generation [29] and myocardial ischemia–reperfusion injury [30]. However, little is known about the role of MYEF2 in tumor. MYEF2 has been confirmed to be a biomarker of smouldering subtypes in the adult T cell leukaemia/lymphoma (ATLL) classification [31]. In addition, MYEF2 is assumed to bind to H1.0 histone mRNA and promote its packaging into extracellular vesicles in melanoma cells, which promotes tumourigenesis [32]. In our current research, we found that MYEF2 could also be a biomarker of GBMs. High expression of MYEF2 indicated better prognosis in GBM patients. Inhibition of MYEF2 expression through siRNA transfection caused promotion of GBM cell proliferation and migration. These findings suggest that MYEF2 might play an important role in GBM progression. Considering the role of MYEF2 in repressing MBP transcription, we inferred that low expression of MYEF2 promoted GBM proliferation and progression through elimination of MBP transcriptional repression.

An increased number of proliferating tumor-reactive CD8+T cells is considered beneficial with respect to a glioblastoma patient’s survival [33]. In our research, along with a negative association between MYEF2 expression and CD8+ T cells infiltration, survival analysis concluded that patients with a low expression of MYEF2 were at a higher risk of poor prognosis. Intriguingly, we observed a negative relation between MYEF2 and several immune-inhibitory factors, like LAG3, PDCD1, CTLA4 TIGIT and HAVCR2, which are characteristics of dysfunctional CD8+ T cells [7]. As a consequence, survival benefits of MYEF2 high expression may be due to the dysfunctional or exhausted states of the CD8+ T cells in GBM. This indicates a beneficial role of MYEF2 in the development of GBM. Thus, MYEF2 may be an effective indicator for the immune microenvironment of GBM. The increased dysfunctional CD8+ T cells was considered as one of the reasons that antagonizing or blocking PD-1 and CTLA-4 have shown only limited efficacy in GBM. More research on the relationship between the decreased MYEF2 expression and resistance to anti-PD1 and CTLA4 therapy may help to improve the efficacy of anti-PD1 and CTLA4 therapy in GBM patients.

## CONCLUSION

In this study, we used the WGCNA and CIBERSORT algorithms to identify CD8+ T cell infiltration-related RBP genes in GBMs. Among the eleven RBP genes being filtered out, we demonstrated that MYEF2 expression was negatively correlated with dysfunctional CD8+ T cells infiltration, and high expression of MYEF2 was associated with better outcomes and lower malignant progression of GBMs. Further molecular, cellular, and animal model studies should be performed to achieve a comprehensive understanding of the mechanism of MYEF2 in immune infiltration and tumor progression in GBMs.

## Supplementary Materials

Supplementary Materials and Methods

Supplementary Figures

Supplementary Tables
