# Supplementary material for: MYEF2: an immune infiltration-related prognostic factor in IDH-wild-type glioblastoma
Source: Aging (Albany NY). 2023 Aug 8;15(15):7760–80. doi: 10.18632/aging.204939 (PMC10457068; doi:10.18632/aging.204939)
Supplement: Supplementary Materials and Methods [file aging-15-204939-s001.pdf]

SUPPLEMENTARY MATERIALS AND METHODS

Gene set enrichment analysis (GSEA)

The samples (*n* = 164) were divided into two groups according to the best cut-off value of myelin expression factor 2 gene (MYEF2) expression (10.5924). The "c2.cp.kegg.v7.0.symbols" gene set was used to carry out enrichment analyses, and a *P*-value < 0.05 and a *q* value < 0.05 were considered statistically significant. The significant pathways were visualized using the R-packages "ggplot2" and "clusterProfiler".

RNA isolation and qRT-PCR

Total RNA was isolated from GBM tissues using TRIzol (Invitrogen), and RNA samples (800 ng per sample) were used to generate cDNA using a PrimeScript™ RT reagent Kit with gDNA Eraser (Takara, Cat. RR047A) according to the manufacturers' instructions. The obtained cDNA samples were used as templates for qPCR amplifications using TB Green® Premix Ex Taq™ II (Tli RNaseH Plus) (Takara, Cat. RR820A). GAPDH was used as a corresponding internal control. All mRNA levels were quantified by the $2^{-\Delta\Delta CT}$ method. Each reaction was performed in triplicate. The primer sequences of MYEF2 were as follows: forward 5′-CAGCTCCAATGGCGTTAAAATG-3′; reverse 5′-TGGCCTTCTTACTTCCTGTAGAT-3′. The primer sequences of GAPDH were as follows: forward 5′-TGACTTCAACAGCGACACCCA-3′; reverse 5′-CACCCTGTTGCTGTAGCCAAA-3′.

GBM tissue single-cell dissociation

GBM tissues were washed with ice-cold Dulbecco's phosphate-buffered saline (DPBS, without Mg^{2+} and Ca^{2+}, Cat.PB180329, Procell, Wuhan) immediately after the operation. Briefly, the samples were dissociated using type IV collagenase (Cat. C8160, Solarbio, Beijing) for 10 min at 37°C. Then, the samples were washed with Dulbecco's modified Eagle's medium (DMEM glucose 4.5 g/L; Biological Industries) and centrifuged (4 min at 300 g, 18°C, minimal braking). The samples were then filtered through a 70 mm cell strainer with DPBS and washed with red blood cell (RBC) lysis buffer (Cat. R1010, Solarbio, Beijing). The dissociated cell suspension was then washed once with DPBS. The cell pellet was resuspended in 1 mL of staining buffer DPBS containing 5% fetal bovine serum (FBS; Biological Industries) and washed one more time.

Immunohistochemistry (IHC)

3 μm sections were mounted on amino propyl ethoxysilane (APES) slides. The slides were deparaffinized, rehydrated, immersed in 10 mM sodium citrate buffer (pH 6.0), pretreated in a microwave oven for 20 min, and then rinsed for 15 min with phosphate-buffered saline (PBS). Endogenous peroxidase was quenched by incubation of the sections in 0.3 % hydrogen peroxide for 30 min at room temperature. Nonspecific binding was blocked by incubation with nonimmune serum (1 % bovine serum albumin for 15 min at room temperature). The sections were incubated overnight with antibody against MYEF2 (rabbit anti-MYEF2, 16051-1-AP, Proteintech, China) at a dilution of 1:200. The next day, the slides were stained with a two-step plus Poly-HRP Anti-Rabbit IgG Detection System (PV-6001; ZSGB-Bio, Beijing, China) to detect MYEF2. After visualization of the reaction with 3,3′-diaminobenzidine, the slides were counterstained with hematoxylin and mounted with synthetic medium.

EdU assays

Cells were plated at a density of 20,000/dish in confocal dishes. After 24 h of incubation, cells were treated with EdU reagent (RiboBio, Cat. C10310-1) for 2 h according to the manufacturer's instructions and then were fixed with 4 % paraformaldehyde. One hundred microliters of 1X Apollo®567 staining reaction solution and Hoechst 33,342 (RiboBio, Cat. C10310-1) was added to each dish and then was incubated for 30 min at room temperature on a decolorization shaker. Cells were then visualized using a BX63 automatic intelligent fluorescence microscope (Olympus, Tokyo, Japan).

In vitro migration assays

Transfected cells were resuspended in serum-free medium, and 200 μl of the cell suspension (5×10^4 cells) was added to the upper millicell chambers (Millipore). The chamber was then cultivated in 5% CO_2 at 37°C for 24 h. Then the cells in the upper chamber were removed, and the attached cells in the lower section were stained with 0.1% crystal violet. The migration rates were quantified by counting the migration cells in five random fields under a light microscope.
