# Supplementary material for: MYEF2: an immune infiltration-related prognostic factor in IDH-wild-type glioblastoma
Source: Aging (Albany NY). 2023 Aug 8;15(15):7760–80. doi: 10.18632/aging.204939 (PMC10457068; doi:10.18632/aging.204939)
Supplement: Supplementary Figures [file aging-15-204939-s002.pdf]

SUPPLEMENTARY FIGURES

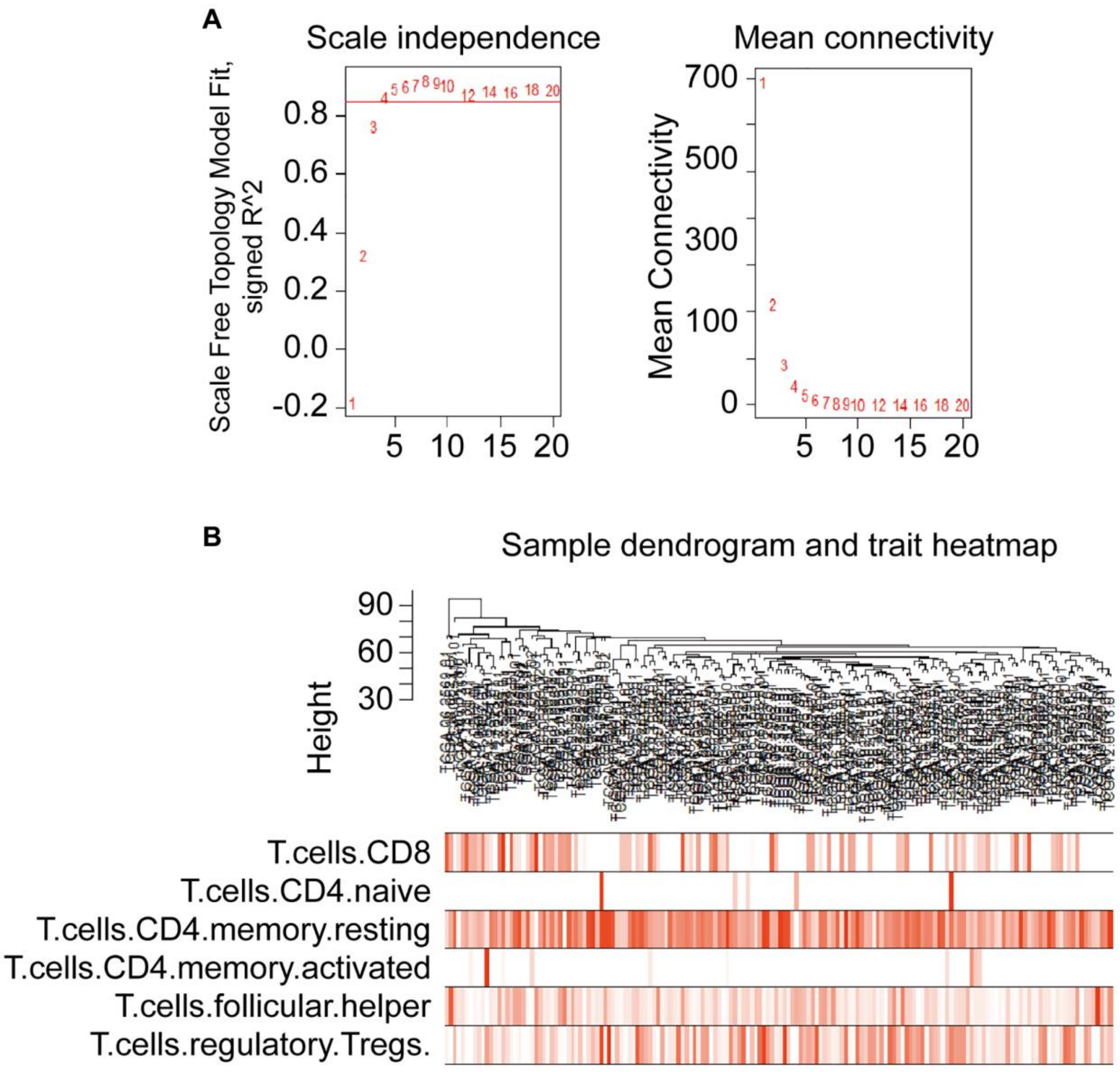

Supplementary Figure 1. Soft-thresholding power analysis and Hierarchical clustering tree of WGCNA analysis. (**A**) Soft-thresholding power analysis including scale-free index and the mean connectivity. (**B**) Hierarchical clustering tree of the 164 TCGA IDH wild-type GBM samples (Value of T cell gamma delta was 0 among all samples).

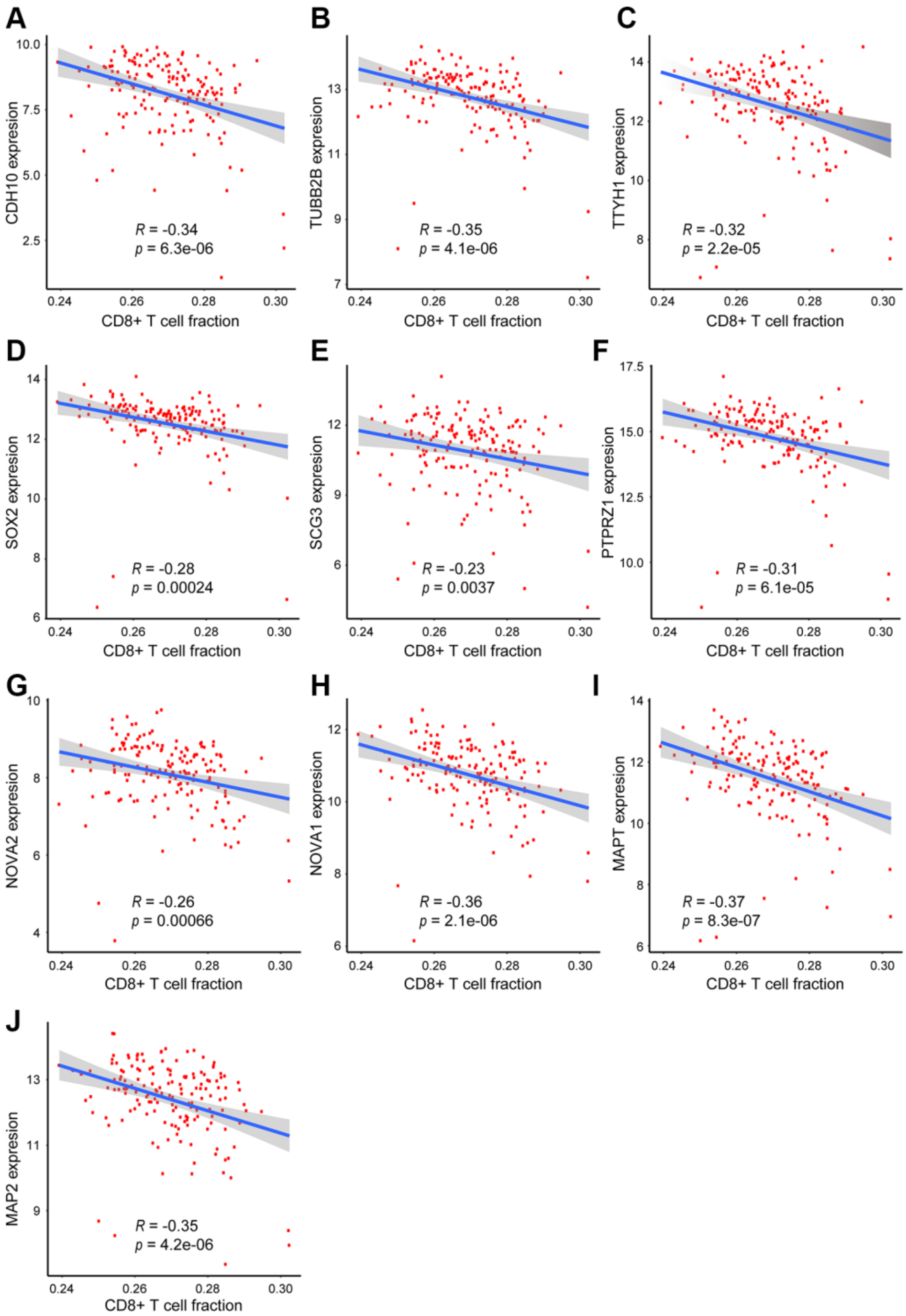

Supplementary Figure 2. (**A**–**J**) Scatter plot of the correlation between hub genes expression and CD8+ T cell infiltration level in TIMER database.

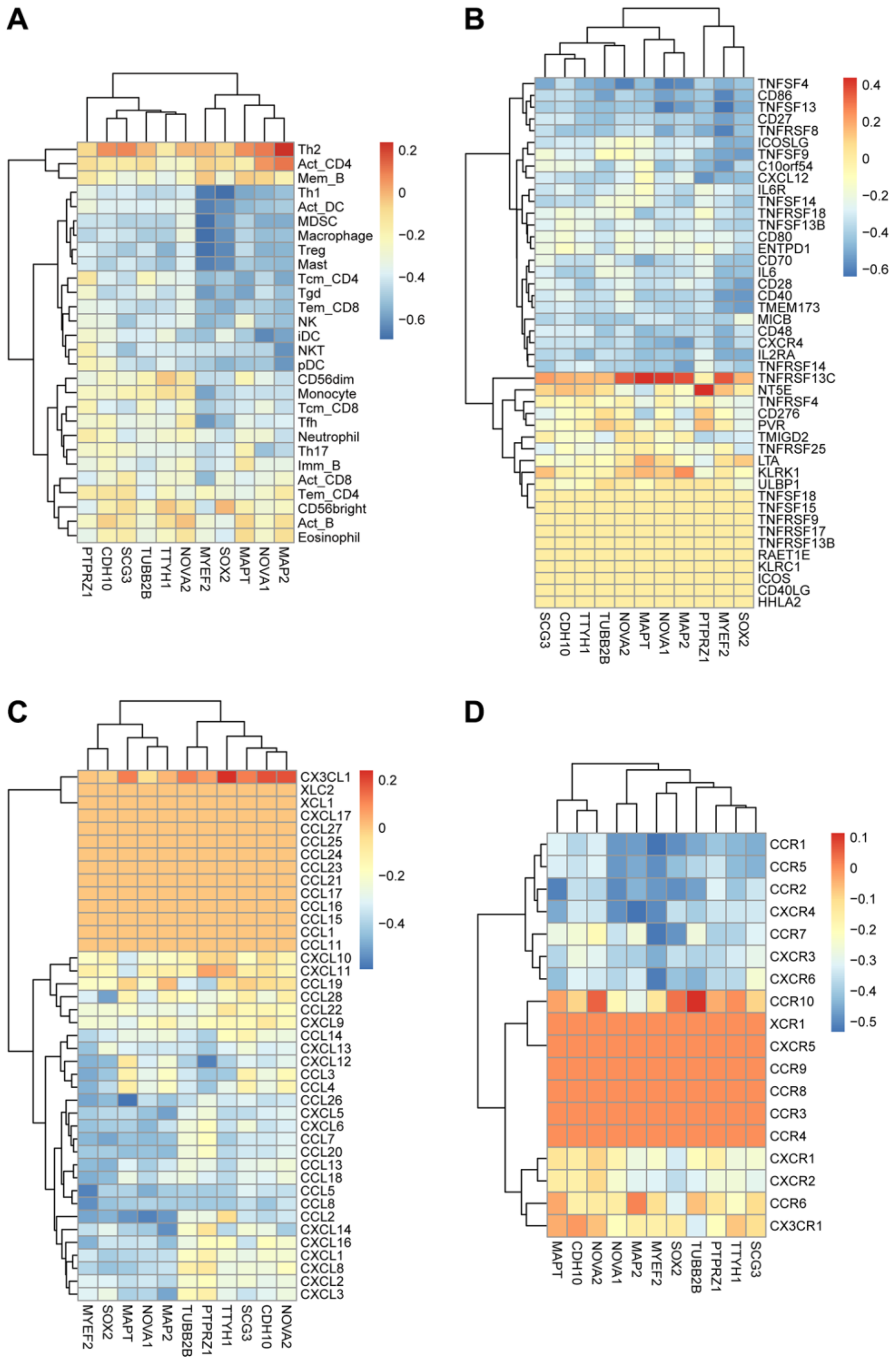

Supplementary Figure 3. Heatmaps of spearman correlations between hub genes expression and TIICs and expression of immune factors in TISIDB database. (**A**) TIICs. (**B**) Immune-stimulatory factors. (**C**) Chemokines. (**D**) Receptors.

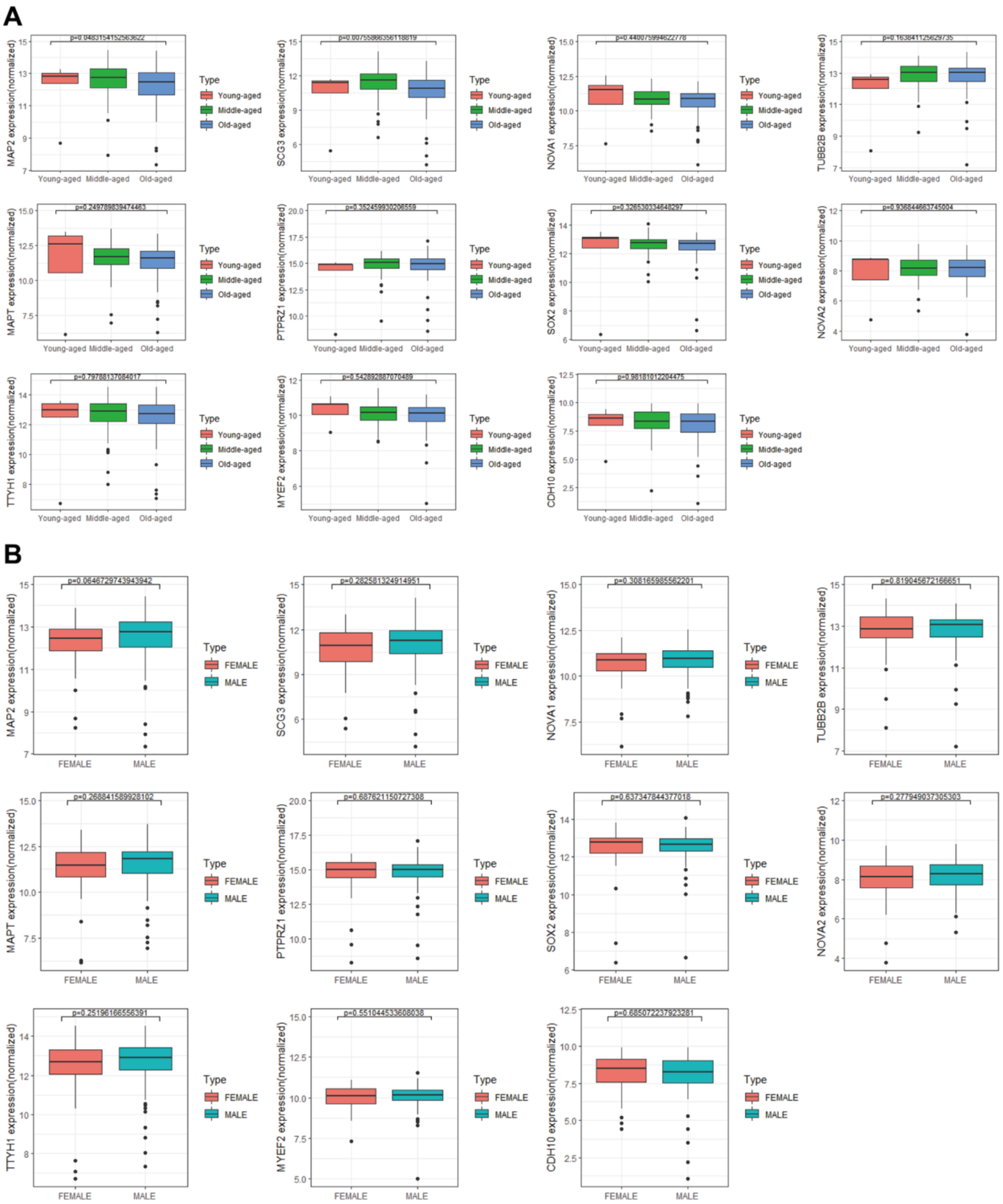

Supplementary Figure 4. Determination of hub genes and clinical characteristics in TCGA database. (**A**) Box plots of hub gene expression in different ages. (**B**) Box plots of hub gene expression in different genders.
