# Supplementary material for: MYEF2: an immune infiltration-related prognostic factor in IDH-wild-type glioblastoma
Source: Aging (Albany NY). 2023 Aug 8;15(15):7760–80. doi: 10.18632/aging.204939 (PMC10457068; doi:10.18632/aging.204939)
Supplement: Supplementary Tables [file aging-15-204939-s003.pdf]

SUPPLEMENTARY TABLES

Supplementary Table 1. Clinical characteristics of IDH wild-type GBM cohort of TCGA database.

	NA (*n* = 10)	Deceased (*n* = 104)	Living (*n* = 49)	Total (*n* = 163)	*P*-Value
**Gender**					
Female	5 (50.0%)	40 (38.5%)	13 (26.5%)	58 (35.6%)	0.2193
Male	5 (50.0%)	64 (61.5%)	36 (73.5%)	105 (64.4%)	
**Age**					
Young-aged (≤30)	1 (10.0%)	1 (1.0%)	3 (6.1%)	5 (3.1%)	
Middle-aged (30–60)	4 (40.0%)	49 (47.1%)	24 (49.0%)	77 (47.2%)	0.2837
Old-aged (>60)	5 (50.0%)	54 (51.9%)	22 (44.9%)	81 (49.7%)	

Supplementary Table 2. Clinical characteristics of the 47 glioblastoma specimens in IHC assay of MYEF2.

Characteristics	MYEF2 expression (According to IHC staining score)		Total (*n* = 47)	*P*-Value
	High (*n* = 21)	Low (*n* = 26)		
**Gender**				
Female	8 (38.1%)	12 (46.2%)	20 (42.6%)	0.5785
Male	13 (61.9%)	14 (53.8%)	27 (57.4%)	
**Age**				
Young-aged (≤30)	2 (9.5%)	3 (11.5%)	5 (10.6%)	
Middle-aged (30–60)	11 (52.4%)	13 (50.0%)	24 (51.1%)	0.9716
Old-aged (>60)	8 (38.1%)	10 (38.5%)	18 (38.3%)	

Supplementary Table 3. Sequences of MYEF2siRNAs.

Sequences of siRNAs	
	Sequence (5′–3′)
siMYEF2 1#	GCAACAUCCCAUAUGACAUTT
siMYEF2 2#	CCCUAGAAACUAUGAACAATT
siMYEF2 3#	CCUGAAGUCAUCAGUAAUUTT
Negative control siRNA	UUCUCCGAACGUGUCACGUTT

Supplementary Table 4. List of candidate hub genes obtained from the green-yellow module of WGCNA.

Candidate hub genes
NOVA1
RAB11FIP1
SEC24D
SOX2
MAP2
MAPT
PTPRZ1
ABCA3
NOVA2

CDH10
CKB
DDR1
MYEF2
NEU4
SCG3
TPM3
TUBB2B
TTYH1
BNC2
